# Low free testosterone is associated with hypogonadal signs and symptoms in men with normal total testosterone levels: results from the European Male Ageing Study

**DOI:** 10.1186/2049-3258-73-S1-P10

**Published:** 2015-09-17

**Authors:** Leen Antonio, Frederick Wu, Terrence O'Neill, Stephen Pye, Emma Carter, Joseph Finn, Michaël Laurent, Ilpo Huhtaniemi, Martin Rutter, Giulia Rastrelli, Gianni Forti, Gyorgy Bartfai, Felipe Casanueva, Krzysztof Kula, Margus Punab, Aleksander Giwercman, Frank Claessens, Brigitte Decallonne, Dirk Vanderschueren

**Affiliations:** 1 KU Leuven, Leuven, Belgium; 2 Andrology Research Unit, University of Manchester, Manchester, United Kingdom; 3 Arthritis Research UK Epidemiology Unit, The University of Manchester, Manchester, United Kingdom; 4 Andrology research Unit, The University of Manchester, Manchester, United Kingdom; 5 Andrology Research Unit, The University of Manchester, Manchester, United Kingdom; 6 Laboratory of Molecular Endocrinology, KU Leuven, Leuven, Belgium; 7 Institute of Reproductive and Developmental Biology, Imperial College London, London, United Kingdom; 8 Andrology Unit, University of Florence, Florence, Italy; 9 Department of Andrology, Albert Szent-Gyorgy Medical University, Szeged, Hungary; 10 Department of Medicine, Santiago de Compostela University, Santiago de Compestela, Spain; 11 Department of Andrology, Medical University of Lodz, Lodz, Poland; 12 Andrology Unit, United Laboratories of Tartu University Clinics, Tartu, Estonia; 13 Reproductive Medicine Centre, Malmö University Hospital, Malmo, Sweden; 14 Laboratory of Clinical and Experimental Endocrinology, KULeuven, Leuven, Belgium

## Background

During ageing, total testosterone (TT) declines and SHBG increases, resulting in a greater decline of free testosterone (FT) compared to TT. However, guidelines suggest using TT to diagnose androgen deficiency and to reserve FT only for men with borderline TT. We investigated if isolated low FT or isolated low TT was associated with androgen-related endpoints in healthy men.

## Methods

3369 community-dwelling men, aged 40-79, were included. We assessed differences between men with both normal TT (=10.5 nmol/L) and calculated FT (=220 pmol/L) (referent), men with normal TT/low FT (group 1) and men with low TT/normal FT (group 2) by descriptive statistics and ordinal logistic regression adjusted for age, centre, BMI and comorbidities.

## Results

2540 men had normal TT (18.4-5.5 [mean-SD] nmol/L) and FT (326-75 pmol/L). There were 261 men in group 1 (normal TT (14.2-3.7 nmol/L), low FT (195-22 pmol/L)) and 92 men in group 2 (low TT (9.6-0.7 nmol/L), normal FT (247-20 pmol/L)).

Compared to referent, men in group 1 were older and had higher SHBG, whereas group 2 was younger and had lower SHBG. Men in group 1, but not group 2, were in poorer health, had lower haemoglobin and a decrease in bone ultrasound measurements. Regression analysis showed that men in group 1 had less frequent morning erections, more erectile dysfunction, fewer sexual thoughts and more physical symptoms (limitations in doing vigorous activity, walking 1 km and bending). Compared to referent, sexual and physical symptoms did not differ in group 2.

## Conclusions

Independent of age, BMI and comorbidities, men with isolated low FT, but normal TT, have more androgen deficiency-related symptoms than men with normal TT and FT levels; whereas symptoms do not differ in men with isolated low TT. Not only total, but also FT levels should therefore be assessed in men with hypogonadal symptoms.

